# Editorial: New strategies for the treatment of advanced melanoma and non-melanoma skin cancer

**DOI:** 10.3389/fmed.2024.1366008

**Published:** 2024-01-23

**Authors:** Alessia Villani, Luca Potestio, Aimilios Lallas

**Affiliations:** ^1^Section of Dermatology, Department of Clinical Medicine and Surgery, University of Naples Federico II, Naples, Campania, Italy; ^2^First Department of Dermatology, School of Medicine, Faculty of Health Sciences, Aristotle University, Thessaloniki, Greece

**Keywords:** melanoma, prevention, skin cancer, dermoscopy, cutaneous tumor

Cutaneous malignancies are classified into melanoma and non-melanoma skin cancers (1). Cutaneous melanoma is responsible for the majority of deaths from skin cancers with survival rates depending on tumor stage at the time of diagnosis, which depends on the depth of the tumor (Breslow), as well as lymph node involvement or distant metastasis (2). Non-melanoma skin cancer includes a heterogeneous group of malignancies including basal cell carcinoma (BCC), cutaneous squamous cell carcinoma (SCC), Merkel cell carcinoma, and cutaneous adnexal tumors (3). Although mortality related to non-melanoma skin cancer is low, due to the high incidence, the absolute number of deaths is comparable to melanoma (2, 3). In the majority of cases these tumors present as localized tumors and are treated with curative surgery or radiotherapy, however, they can also present in an advanced or metastatic stage thus requiring systemic treatments. In the last decade, the therapeutic landscape has recently expanded with the development of immunotherapy and targeting therapies. Hence, the update provided in this Research Topic on cutting-edge topics such in the management of advanced cutaneous malignancies is helpful to offer readers a wide perspective.

Globally, surgery is the mainstay of treatment for skin cancers. However, the application of surgery is occasionally challenging, such as in tumors located on difficult-to-treat areas, very advanced tumors or tumors with poorly defined clinical margins (4). In this context, Mohs micrographic surgery represents the optimal modality to ensure a complete resection with free-of-disease margins (5). However, this technique is limited by high costs. Surmanowicz et al. reported the efficacy and safety of the muffin technique micrographic surgery (MTMS), an alternative micrographic technique wherein the entire excised margin is evaluated post-operatively by a pathologist using paraffin-embedded material. The authors reported no cases of tumor recurrence after MTMS approach (Surmanowicz et al.).

In locally advanced tumors, surgery might be extremely complicated or even impossible. Recent knowledge on skin cancer pathogenesis led to the development of selective and effective drugs. In this scenario, Rubatto et al. offered a wide perspective reporting classic and new strategies for the treatment of advanced melanoma and non-melanoma skin cancer. Regarding non-melanoma skin cancer, the comprehension of the aberrant activation of the hedgehog signaling pathway in basal cell carcinoma led to the development of hedgehog inhibitors: sonidegib and vismodegib (Rubatto et al.). Immunotherapy has showed to be effective in the management of melanoma, SCC and Merkel cell carcinoma (Rubatto et al.). Targeting PD1/PDL1 (nivolumab, pembrolizumab, and avelumab) or CTLA4 (ipilimumab) signaling pathway seem to be a useful weapon to reduce the immune escape strategy adopted by cancer (Rubatto et al.). Of interest, cemiplimab has been licensed for advanced BCC unresponsive to hedgehog inhibitors (6). Moreover, targeted therapy, particularly anti-BRAF (dabrafenib, vemurafenib, and encorafenib) and anti-MEK (trametinib) showed to be effective in melanoma with specific genetic mutation (Rubatto et al.). Finally, several strategies such as treatment combination or their use in neoadjuvant setting as well as several new drugs (e.g., intravenous oncolytic virus, antivascular endothelial growth factor, etc.) are under investigations (Rubatto et al.). In this context, Grätz et al. reported the results of a retrospective study in 11 patients with metastatic melanoma to assess whether immune checkpoint- or targeted therapy should be commenced up front, the optimal time for changing treatment, specifically to prevent resistance whilst maintaining disease control. Although the authors reported that elective switching from targeted to immune checkpoint therapy was associated with a better survival outcome, it is unclear whether the choice of initial therapy confers long–term survival and disease-control advantages (Grätz et al.).

Finally, also the emerging role of radiotherapy (RT) has been investigated. In particular, Benkhaled et al. highlighted the potential use of RT in non-melanoma skin cancer when surgery is not an option or will cause unacceptably functional morbidity. Globally, brachytherapy, contact hypo fractionated RT, and electronic brachytherapy are promising techniques. However, rigorous clinical trials are missing (Benkhaled et al.). Of interest, RT can also be used in patients with advanced melanoma, despite melanoma is a radioresistant cancer (Sabbah et al.). Indeed, as reported by Sabbah et al., RT induces intracellular translocation of receptor tyrosine kinase (RTK), which regulates response to DNA damage activating proteins and promotes DNA repair. As a consequence, the authors hypothesized that co-targeting DNA repair (PARP-1) and relevant activated RTKs, may radiosensitize wild-type B-Raf Proto-Oncogene, Serine/Threonine Kinase (WTBRAF) melanomas where RTKs are often upregulated (Sabbah et al.). Therefore, a synergistic effect is auspicable, not only on tumor growth inhibition but also on tumor regrowth control (Sabbah et al.). However, further studies are required.

Overall, the management of advanced skin cancers is moving toward a personalized approach. A better comprehension of available drugs, better selection of individuals that might benefit from each drug, indications for combined treatments, represent important challenges that, if addressed, may improve patients' prognosis. Moreover, several drugs currently under investigation will allow to offer treatments to patients unresponsive to currently available therapies. Undoubtedly, these selective new drugs replaced the conventional chemotherapeutic agents, offering to patients affected by advanced cutaneous malignancies a more personalized approach. However, there are many points to be addressed and more studies are required.

## Author contributions

AV: Writing – original draft, Writing – review & editing. LP: Writing – original draft, Writing – review & editing. AL: Writing – original draft, Writing – review & editing.

## In Memoriam

In memory of Professor Gabriella Fabbrocini.
